# Primary Intramedullary Malignant Lymphoma in the Cervical Cord with a Presyrinx State

**DOI:** 10.7759/cureus.2006

**Published:** 2017-12-30

**Authors:** Kohei Chida, Atsushi Sugawara, Takahiro Koji, Takaaki Beppu, Yoshiharu Mue, Tamotsu Sugai, Knuaki Ogasawara

**Affiliations:** 1 Neurosurgery, Iwate Medical University; 2 Molecular Diagnostic Pathology, Iwate Medical University

**Keywords:** primary malignant lymphoma, intramedullary, presyrinx state

## Abstract

A 79-year-old man presented with primary intramedullary malignant lymphoma with a presyrinx state in the cervical cord manifesting as left hemiparesis and hemidysesthesia. The magnetic resonance imaging (MRI) scan showed an intramedullary mass in the cervical spinal cord at the level of C1 and T2-weighted image prolongation from the medulla to the level of C5. According to the progression of hemiparesis, he underwent an emergency removal of the tumor under general anesthesia. The tumor was totally removed, and the peritumoral signal abnormality was not present in the postoperative MRI. Histological examination revealed diffuse large B cell lymphoma. While brain MRI, bone marrow puncture, and ^18^F-fluorodeoxy-glucose positron emission tomography (18FDG-PET) of the whole body were performed to find out a primary lesion, there were no abnormalities. He underwent a high-dose methotrexate-based chemotherapy and a local irradiation therapy (40Gy). He has been alive for more than two years since the symptom onset, and without any evidence of recurrence. This case suggests that malignant lymphoma, as an infiltrating and rapidly progressive tumor, may be accompanied by syrinx.

## Introduction

A presyrinx state may develop from a nontraumatic obstruction of the cerebrospinal fluid (CSF) pathways in the spine [1]. While the pathogenesis resolves after restoring the patency of the CSF pathways, a frank syrinx can result if left untreated [1]. Infiltrating and rapidly progressive tumors such as malignant lymphoma are less likely to be accompanied by syrinx [2]. We report the rare case of a primary intramedullary malignant lymphoma with a presyrinx state in the cervical cord.

## Case presentation

**Figure 1:**
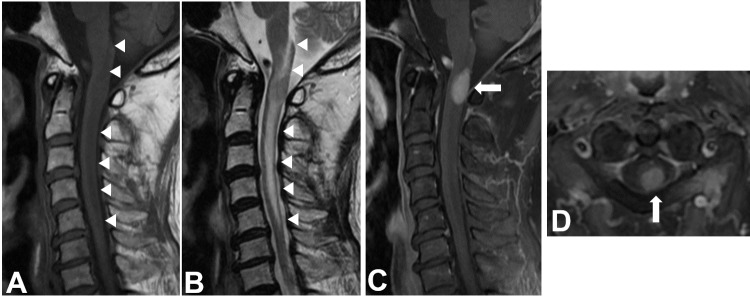
Preoperative magnetic resonance imaging Sagittal magnetic resonance imaging (MRI) showing an intramedullary mass in the cervical cord at the level of C1 associated with syringomyelia expanding from the medulla to the level of C5 (arrowhead). The lesion appears as an isointense mass on the T1 and T2-weighted images (A and B) and as a homogeneously-enhanced mass with gadolinium (C) (arrow). Axial MRI showing a mass lesion largely displacing from the spinal cord (D) (arrow).

A 79-year-old man presented with a six-month history of numbness in his left upper limb. Left motor weakness developed suddenly, and the patient was admitted to our hospital. Neurological examination on admission revealed left hemiparesis with 2/5 in his upper limb and 3/5 in his lower limb and hypoesthesia in his left side from the neck to the foot. The deep tendon reflexes were increased in his left upper limb. Magnetic resonance imaging (MRI) showed an intramedullary tumor that was homogeneously enhanced with gadolinium at the C1 level in the spinal cord (Figure 1). The tumor was also surrounded by a signal change that appeared isointense to the CSF and was clearly differentiated from the spinal cord (Figure 1). The signal change seemed to represent a presyrinx state.

The patient underwent removal of the tumor under general anesthesia. Laminectomy of the C1 and C2 levels was performed, combined with a suboccipital craniotomy. The tumor was located intramedullary, and the border between the tumor and the neural tissue was unclear. It also invaded into the dorsal root of the C1, and the root was removed with the tumor.

**Figure 2:**
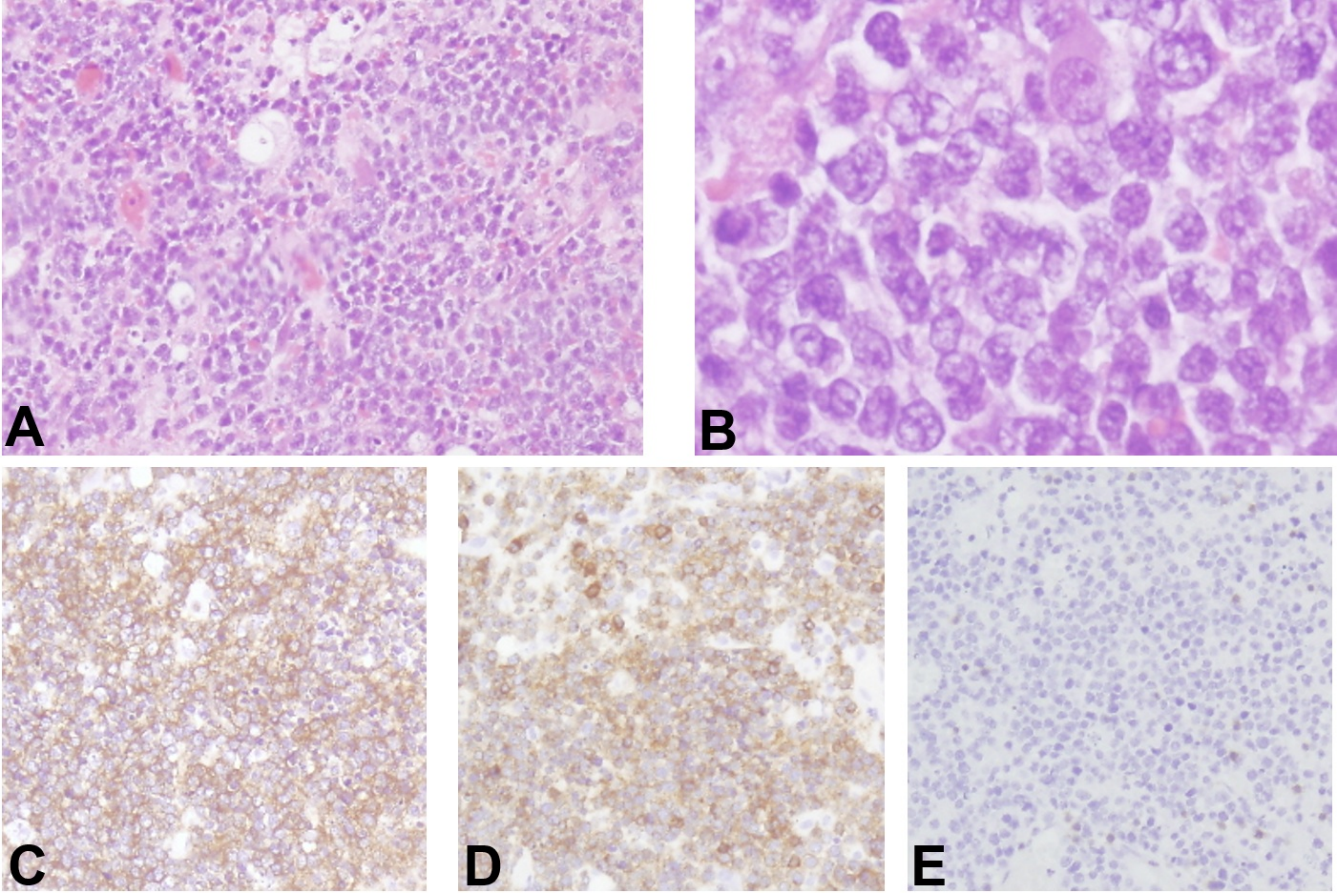
Histological examinations Photomicrographs of the tumor showing diffuse proliferation of large atypical lymphocytes (A: hematoxylin and eosin stain, original magnification ×100, B: hematoxylin and eosin stain, original magnification ×400), which were positive for CD20 (C: immunohistochemical staining for CD20) and CD79a (D: immunohistochemical staining for CD79a), and negative for CD3 (E: immunohistochemical staining for CD3).

**Figure 3:**
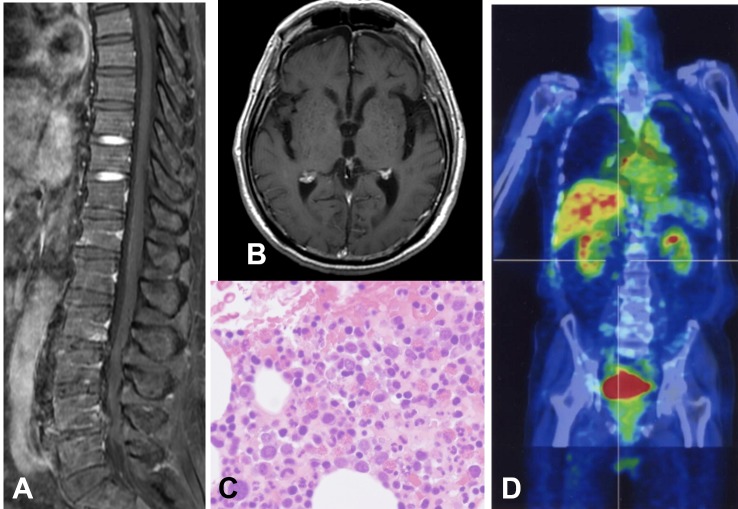
Other examinations Magnetic resonance imaging (MRI) of the lumbar spine and brain (A and B), bone marrow puncture (C) and ^18^F-fluorodeoxy-glucose positron emission tomography (18FDG-PET) of the whole body (D) showing no other primary lesion.

**Figure 4:**
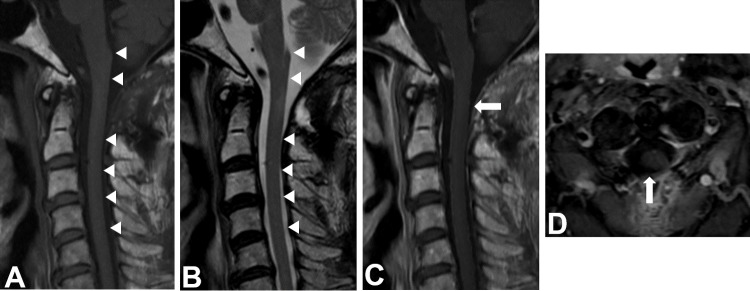
Postoperative magnetic resonance imaging Magnetic resonance imaging (MRI) performed three months after surgery showing no evidence of tumor recurrence and disappearance of the syringomyelia (A, B, C, and D) (arrow and arrowhead).

Postoperatively, his motor function recovered to the level of 3/5 in his upper limb and 4/5 in his lower limb. Histological examination of the tumor revealed a diffuse proliferation of large atypical lymphocytes with B cell markers (Figure 2). Based on these findings, the tumor was diagnosed as diffuse large B cell lymphoma. A subsequent MRI of the other regions, a bone marrow puncture, and an ^18^F-fluorodeoxy-glucose positron emission tomography (18FDG-PET) of the whole body showed no abnormalities (Figure 3). The patient underwent high-dose methotrexate (MTX)-based chemotherapy and localized external beam radiation therapy (40Gy) for the level of C1 in the cervical cord. An MRI performed three months postoperatively showed total removal of the tumor and complete disappearance of the peritumoral signal abnormality (Figure 4).

 

 

He has been alive for more than two years since the symptom onset, and without any evidence of recurrence.

Written informed consent was obtained from the patient and his next of kin prior to any procedures.

## Discussion

Transudation from pathological tumor vessels, secretion by tumor cells, and obstruction of the CSF flow by an intramedullary tumor might be significant factors for the development of syringomyelia. Displacing tumors are more likely to cause syrinxes than infiltrating tumors [2]. Nakamizo, et al. reported malignant lymphoma is less likely to be accompanied by syrinx because of its infiltrating and rapidly progressive nature [3]. To our knowledge, only two patients of primary intramedullary malignant lymphoma with syringomyelia have been reported previously. One case was demonstrated on MRI, but the other was detected on the necropsy [4-5]. In our case, malignant lymphoma infiltrated horizontally which may have disturbed the CSF flow and eventually led to a syrinx.

A presyrinx state has been defined as a spinal cord parenchymal T2 prolongation that is reversible after restoration of patency of the CSF pathways [1]. It may cause minimal clinical symptoms or even be asymptomatic. In our case, since parenchymal abnormal signals had completely recovered after treatment, the lesion should be categorized as a presyrinx state.

The most important steps in the approach to spinal cord lymphoma are biopsy and histological diagnosis followed by chemotherapy [6]. MTX was the mainstay of initial therapy, but relapse was common. So additional treatments with chemotherapy regimen used in the treatment of non-Hodgkin lymphoma (R-CHOP), radiation, or peripheral blood stem cell transplantation were necessary in most patients [7-8]. In our case, however, we decided to remove the tumor rather than perform a biopsy for two reasons. One was that the patient’s paralysis progressed so rapidly that mass reduction was necessary. The other reason was that the MRI findings did not show malignant lymphoma associated with syringomyelia. In consequence, the patient’s motor function was recovered after surgery, and postoperative chemotherapy and radiation therapy led to a clinically stable state and radiologic improvement.

## Conclusions

This case suggests that malignant lymphoma, as an infiltrating and rapidly progressive tumor, can be accompanied by syrinx. With this in mind, intraoperative rapid pathologic diagnosis before resection should be considered for intramedullary spinal tumors with syrinx.
